# Optimization of descriptors and cross-references across the different versions of the German National Competence Based Catalogue of Learning Objectives for Undergraduate Medical Education (NKLM)

**DOI:** 10.3205/zma001809

**Published:** 2026-02-17

**Authors:** Till Rech, Jacqueline Jennebach, Martin R. Fischer, Felix Balzer, Firman Sugiharto, Martin Dittmar, Vincent Wyszynski, Olaf Fritze, Simon Drees, Olaf Ahlers

**Affiliations:** 1 Charité – Universitätsmedizin Berlin, Institute of Medical Informatics, LOOOP Research Team, Berlin, Germany; 2 Charité – Universitätsmedizin Berlin, Department of Neonatology, Berlin, Germany; 3 German Association of Medical Faculties, Berlin, Germany; 4 LMU University Hospital, LMU Munich, Institute of Medical Education, Munich, Germany; 5 Duke-NUS Graduate Medical School, Department for Technology-Enhanced Learning and Innovation, Singapore; 6 Charité – Universitätsmedizin Berlin, IT Department, Berlin, Germany; 7 Charité – Universitätsmedizin Berlin, Department of Anesthesiology and Intensive Care Medicine CCM/CVK, LOOOP Research Team, Berlin, Germany; 8 University of Tübingen, Medical Faculty, TIME - Tübingen Institute for Medical Education, Tübingen, Germany; 9 Brandenburg Medical School Theodor Fontane, Faculty of Health Sciences Brandenburg, Institute of Research in Health Sciences Education, Neuruppin, Germany

**Keywords:** curriculum, competency-based medical education, frameworks, LOOOP

## Abstract

**Background:**

The National Competence Based Catalogue of Learning Objectives for Undergraduate Medical Education (NKLM) has been developed as a curricular framework since 2009 and was published in version 1.0 in 2015. Refining the clinical disease descriptors and their associated cross-references (CR) is necessary to improve the clarity and usability of the NKLM. This study examines whether this optimization could be achieved through targeted interventions during the further development of the NKLM 1.0 via the intermediate versions NKLM 1.0 (neo) and NKLM 2.0 to NKLM 2.1..

**Methods:**

The revision of the NKLM was supported by structured interventions such as the conversion of free text into CR, the introduction of essential and non-essential CR, and the linking of clinical descriptors exclusively to selected learning objective chapters. Subsequently, an analysis was performed to determine whether the number of descriptors and associated CR had been reduced and whether the clinical competencies were completely specified.

**Results:**

The number of clinical descriptors set per disease was significantly reduced (by 29% for diagnostics, 17% for therapy, 75% for emergency measures, and 66% for prevention/rehabilitation). The number of CR was also significantly reduced. At the same time, all existing gaps in the network (consisting of clinical descriptors and associated CR for learning objectives) were closed.

**Conclusion:**

The interventions resulted in a clearer network of descriptors and CR, providing a more precise definition of the content to be learned. This means that the NKLM can presumably better fulfill its purpose with regard to the constructive alignment of learning, teaching, and assessment.

## Zusammenfassung

**Hintergrund:**

Der Nationale Kompetenzbasierte Lernzielkatalog Medizin (NKLM) wurde seit 2009 als curriculares Rahmenwerk entwickelt und 2015 in der Version 1.0 veröffentlicht. Eine Optimierung der klinischen Erkrankungs-Deskriptoren und der zugehörigen Querverbindungen (QV) im Sinne einer präziseren Definition der zu erlernenden Inhalte ist essenziell, um die Handhabbarkeit und Übersichtlichkeit des NKLM zu verbessern. Die vorliegende Arbeit untersucht, ob diese Optimierung durch gezielte Interventionen während der Weiterentwicklung vom NKLM 1.0 über die Zwischenversionen „NKLM 1.0 (neo)“ und „NKLM 2.0“ zum „NKLM 2.1“ erreicht werden konnte.

**Methoden:**

Die Überarbeitung des NKLM wurde unterstützt durch strukturierte Interventionen wie die Umwandlung von Freitext in QV, die Einführung essenzieller und nicht-essenzieller QV sowie die Verbindung klinischer Deskriptoren ausschließlich mit ausgewählten Lernzielkapiteln. Im Anschluss wurde analysiert, ob sich die Zahl der Deskriptoren und der zugehörigen QV reduziert hat und ob eine lückenlose Definition der zu erlernenden klinischen Kompetenzen erfolgt ist.

**Ergebnisse:**

Die Zahl der gesetzten klinischen Deskriptoren pro Erkrankung wurde signifikant reduziert (bei Diagnostik um 29%, bei Therapie um 17%, bei Notfallmaßnahmen um 75% und bei Prävention/Rehabilitation um 66%). Auch die Zahl der QV konnte signifikant reduziert werden. Gleichzeitig wurden alle vorliegenden Lücken im Netzwerk (aus klinischen Deskriptoren und zugehörigen QV zu Lernzielen) geschlossen.

**Schlussfolgerung:**

Mit Hilfe der vorgenommenen Interventionen entstand ein übersichtlicheres Netzwerk aus Deskriptoren und QV, das die zu erlernenden Inhalte präziser definiert. Damit kann der NKLM seinen Nutzen im Hinblick auf das *constructive alignment* von Lernen, Lehren und Prüfen mutmaßlich besser erfüllen.

## 1. Introduction

### 1.1. Background

Internationally, it is recommended that medical curricula are mapped to overarching frameworks that define the competencies to be acquired by students [1], [2], [3]. Among other things, this enables the so-called *constructive alignment* of learning, teaching, and assessment as a central quality criterion for outcome- and competency-based curricula [4]. The National Competence Based Catalogue of Learning Objectives for Undergraduate Medical Education (NKLM) was developed under the auspices of the German Society for Medical Education (GMA) and the German Association of Medical Faculties (MFT) and published in version 1.0 in 2015. This national framework defines the competencies that medical students in Germany are expected to acquire [5], [6], [7]. As part of a German master plan for Medical Education [8], the NKLM was further developed by approximately 800 experts with a view to future binding force by a new Medical Licensing Regulation (ÄApprO). This development was carried out in coordination with the development of a competence-oriented catalogue for the German national state exams. It has been published in 2021 as NKLM 2.0 [9]. Based on a standardized evaluation process involving 38 medical faculties [10], a further revision to version 2.1 was started in 2022. Due to the complexity of the revision process, it was divided into two phases. Approximately 50% of NKLM 2.0 was revised by 100 experts in eleven “focus groups” (FG) to create version 2.1. The revision process for this version has progressed sufficiently to allow the analyses presented here. The NKLM, including the remaining 50%, will be revised to version 3.0 after the release of version 2.1.

Ease of use and clarity are essential both for the revision process described above and for the subsequent use and acceptance of the NKLM in medical faculties [11]. For example, for each disease, it should be clearly identifiable which therapeutic procedures students are expected to learn [1]. The revision of the NKLM has therefore been taking place since 2018 as part of a collaboration with the LOOOP network for Research in Health Sciences Education, which has been developing concepts for curricular mapping since 2004 [12], [13], [14] and has been jointly coordinated by Charité – Universitätsmedizin Berlin and Brandenburg Medical School Theodor Fontane (MHB) since 2023. This network has incorporated the LOOOP concepts into the NKLM and provided an online platform for the NKLM revision.

### 1.2. Chapters and cross-references within the NKLM

The NKLM consists of various chapters (sections) that either list keywords or learning objectives (LO). References between chapters are established via links called cross-references (CR). CR are possible between all chapters.

The following section introduces the *chapters and CR relevant to the context of this work*. The terms, some of which have been renamed in the course of the various catalogue versions, are used in accordance with the latest catalogue version, NKLM 2.1.

#### 1.2.1. Diseases and associated descriptors

The chapter “diseases” contains a list of diseases for which competencies are to be acquired. These competencies are defined using six so-called descriptors. Four of these descriptors correspond to learning objectives formulated in other chapters and are therefore the subject of this paper:

1. Diagnosis (D)
2. Therapy (T)
3. Emergency measures (N)
4. Prevention/ Rehabilitation (P)

For each descriptor, the depth of competence (hereinafter referred to as “depth”) is used to define whether, during the course of study,

- *Knowledg*e (K) or
- *Competence to act* (A)

is to be acquired during the course of study (example: [https://nklm.looop-network.org/objective/list/objective/10006348]).

#### 1.2.2. Learning objectives and associated study sections

Formulated LO are listed in the NKLM embedded in (partial) competencies. These LO also represent different depths, with a finer granularity than the descriptors. Both the K category and the A category consist of two possible depths each, resulting in a total of four possible depths:

- Factual *knowledge* (depth 1),
- Acting and reasoning *knowledge *(depth 2),
- *Competence to act *under supervision (depth 3a),
- *Competence to act *unsupervised (depth 3b).

These depths are assigned to one or more study sections, defining which aspects of a long-term project should be learned in which study section at the latest (example: [https://nklm.looop-network.org/objective/list/objective/10006728]). The rationale behind this definition of study sections is the above mentioned* constructive alignment* with the state exams that conclude the study sections.

A group of LO covers the content of the above-mentioned clinical sub-aspects D, T, N, and P. The four chapters in which these LO are located are referred to below as “target chapters”.

#### 1.2.3. Cross-references between diseases and clinical learning objectives

The assignment of set descriptors to specific content is done via CR between the respective disease and the corresponding clinical LO. This CR defines which content should be learned in what depth and by which stage of study at the latest. On the one hand, this means that each set descriptor must be assigned at least one LO with sufficient depth of competence. So, for example, if the descriptor D defines a competence to act, at least one diagnostic learning objective with competence to act must also be cross-linked. This interaction between descriptors and learning objectives is referred to below as “congruence”. On the other hand, the number of descriptors set and thus the associated CR must take into account that the content mapped in this way (“network”) has to be implemented in a local curriculum in context with each other (i.e., usually in the same courses).

#### 1.2.4. Structural features of the NKLM versions

*NKLM 1.0:*

- For each disease, the four descriptors were implicitly assigned with at least the level Knowledge (K). Only the descriptor Competence to act (A) was explicitly marked.
- References between chapters could be established via two free text categories (1. “examples”, 2. “reasons for consultation/diseases”) that were not cross-referenced to other NKLM content.

This made navigation and thus the usability of the catalogue more difficult, which in turn reduced the urgently needed acceptance of the work [11], [15].

*NKLM 2.0 and 2.1*

- Each descriptor had to be set individually.
- References between chapters were established exclusively via CR.

### 1.3. Aim of this work and questions

Based on NKLM 1.0, the total number of descriptors and CR was to be reduced through gradual interventions. The aim was to create a coherent and homogeneous network of disease descriptors and cross-referenced clinical LO (CR-LO). This aligns with the recommendation made by the authors of the 2015 NKLM. *Completing the cross-references and reviewing the interaction between diseases and the other parts of the NKLM in particular will be an important part of the trial phase under the responsibility of the medical faculties* [6].

In addition, the following questions should be answered:

- How many cross-references can be generated by converting the free text of NKLM 1.0?
- How does the number of descriptors change across the different catalogue versions?
- How does the number of cross-references change, and how completely is the network of descriptors and associated cross-references represented in the different catalogue versions?

## 2. Methods

### 2.1. Step 1 (2016): Conversion of free text into cross-references for NKLM 1.0 (neo)

The references in NKLM 1.0, which were only available as free text (often as lists), were first divided into meaningful individual entries and then converted into CR (“mapping”) if there was matching, referable content in the catalogue. First, a pilot assignment was carried out using 280 examples. Based on this experience, rules were developed inductively for the subsequent mapping of the entire data set:

- No additional related topics were introduced; cross-references were created only when the existing term matched an existing item in the catalogue.
- Entries were only mapped if all CR (in total) could achieve complete coverage of the term’s content.

Based on these rules, all references available in text form were then mapped. To reduce the workload, the catalogue was divided into chapters between authors TR and SD. For each CR, it was noted whether it was an original CR (links category 1), a previous “example” (links category 2), or a previous entry under “reasons for consultation/diseases” (links category 3). Step 1 was carried out by the LOOOP research team before the start of the cooperation with the MFT. The supplemented NKLM version is referred to below as NKLM 1.0 (neo) [https://nklm-10-neo.looop-network.org].

### 2.2. Step 2 (2018-2021): Transfer of results and development of NKLM 2.0

The NKLM 1.0 (neo) formed the basis for the development of the NKLM 2.0. One goal of this development was to reduce the total number of descriptors set per disease and also the number of descriptors with competence to act. The FG experts therefore critically and systematically reflected on which aspects of a disease should be learned by all students nationwide and where competence to act actually needs to be acquired during their studies. In some cases, larger disease groups were divided up in order to be able to assign more differentiated descriptors. In addition, there was a requirement to assign at least one corresponding LO to each descriptor (“essential” CR) in order to correctly map the network. Furthermore, the necessity of the pre-existing CR between all other chapters was to be critically reflected upon.

### 2.3. Step 3 (since 2022): Development of the NKLM 2.1

Since many experts still considered the number of CR to be too extensive, the CR were divided into two groups: Group 1 contained only those CR that are indispensable for the usability of the NKLM. This was primarily the portion of the “essential” QVs already described that had a direct connection to D, T, N, or P. while all other CR were defined as “non-essential” and deactivated throughout the NKLM. Only essential CR were revised in terms of content by the FG experts for NKLM 2.1.

For the diseases, a mandatory link between a set descriptor and the corresponding target chapter was now specified, although it was still possible to create several CR for a descriptor.

Furthermore, it was defined that general medical history taking and physical examination are automatically required for each disease and therefore do not require a descriptor marking or a CR for the learning objectives that remain in the catalogue.

### 2.4. Analyses

For step 1, an analysis was performed of the CR available for all three reference categories. Since some CR in reference category 1 in NKLM 1.0 had been created in only one direction (unidirectional) and others bidirectionally (i.e., the same CR existed twice), all CR were created bidirectionally for the analysis in order to standardize them. In addition, the number of free texts that could not be converted into CR was determined. Subsequently, the absolute number of bidirectional CR before and after mapping was determined and visualized for all chapters using cross tables.

For the subsequent analyses, only essential CR were considered unidirectionally: The number of diseases, descriptors, and assigned CR were evaluated for the various catalogue versions after the interventions had been carried out. The completeness of the network of descriptors and associated LOs was also analysed. For this purpose, two variants were calculated for NKLM 1.0 (neo) and NKLM 2.0: Variant 1 only took into account references to the target chapters later defined as such. Variant 2 also took into account references to other chapters.

Chi-square tests were performed to determine significant differences (defined by a p<0.05) between the catalogue versions. All calculations were performed in Excel (version 2411) or R (version 4.4.1 [https://www.R-project.org/]).

## 3. Results

### 3.1. Conversion of free text into cross-references for NKLM 1.0 (neo)

**Figure 1:**
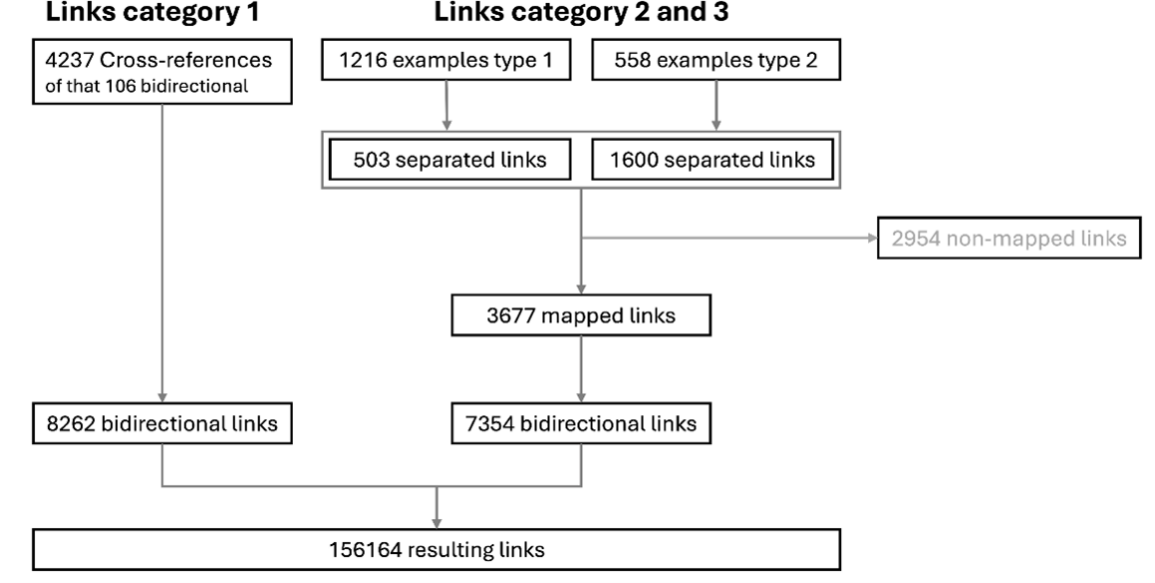
Representation of the number of links before and after the first intervention Some of the “examples” (links type 2) and “reason for consultation/diseases” (links type 3) could not be mapped because there was no corresponding content in NKLM 1.0

55% of the CR (categories 2 and 3) created by separating the term lists into individual terms could be mapped against other NKLM entries. Overall, this process step led to a 368% increase in CR. Figure 1 (Fig. 1) shows the absolute numbers.

**Figure 2:**
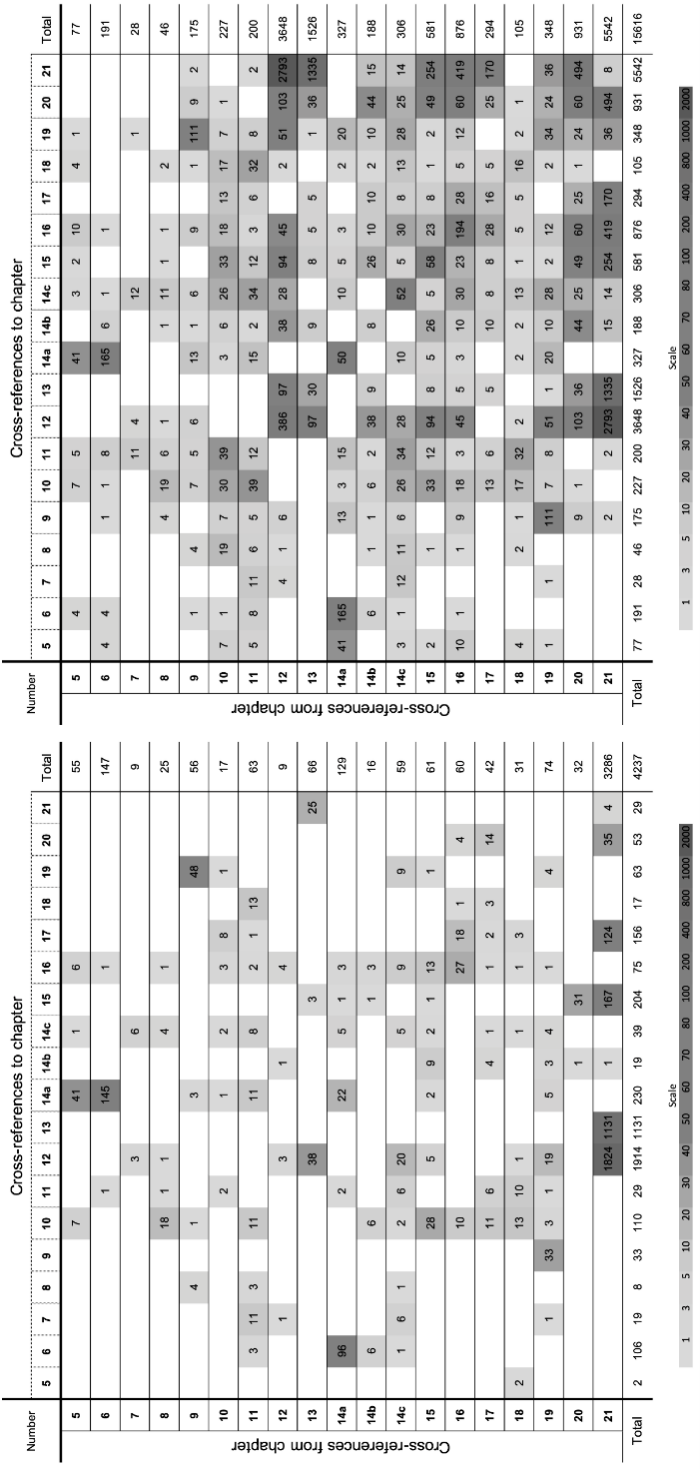
Cross tables of cross-connections between chapters before and after the first intervention, NKLM 1.0 on the left, NKLM 1.0 (neo) on the right Cross-references between all relevant chapters are shown. The brightness scale at the bottom shows the logarithmic correlation of the brightness levels with the number of cross-references

The CR between the chapters of NKLM 1.0 and NKLM 1.0 (neo) are plotted in figure 2 (Fig. 2). There is a clear increase in the density of the (also bidirectional) CR, although the chi-square test continues to show an inhomogeneous distribution in NKLM 1.0 (neo). NKLM 1.0: p<0.001/Cramer’s V: 0.513, NKLM 1.0 (neo): p<0.001/Cramer’s V: 0.387.

### 3.2. Number of descriptors used

The number of diseases represented in the NKLM varies across catalogue versions:

- NKLM 1.0/NKLM 1.0 (neo): 441 diseases
- NKLM 2.0: 598 diseases
- NKLM 2.1: 555 diseases

**Figure 3:**
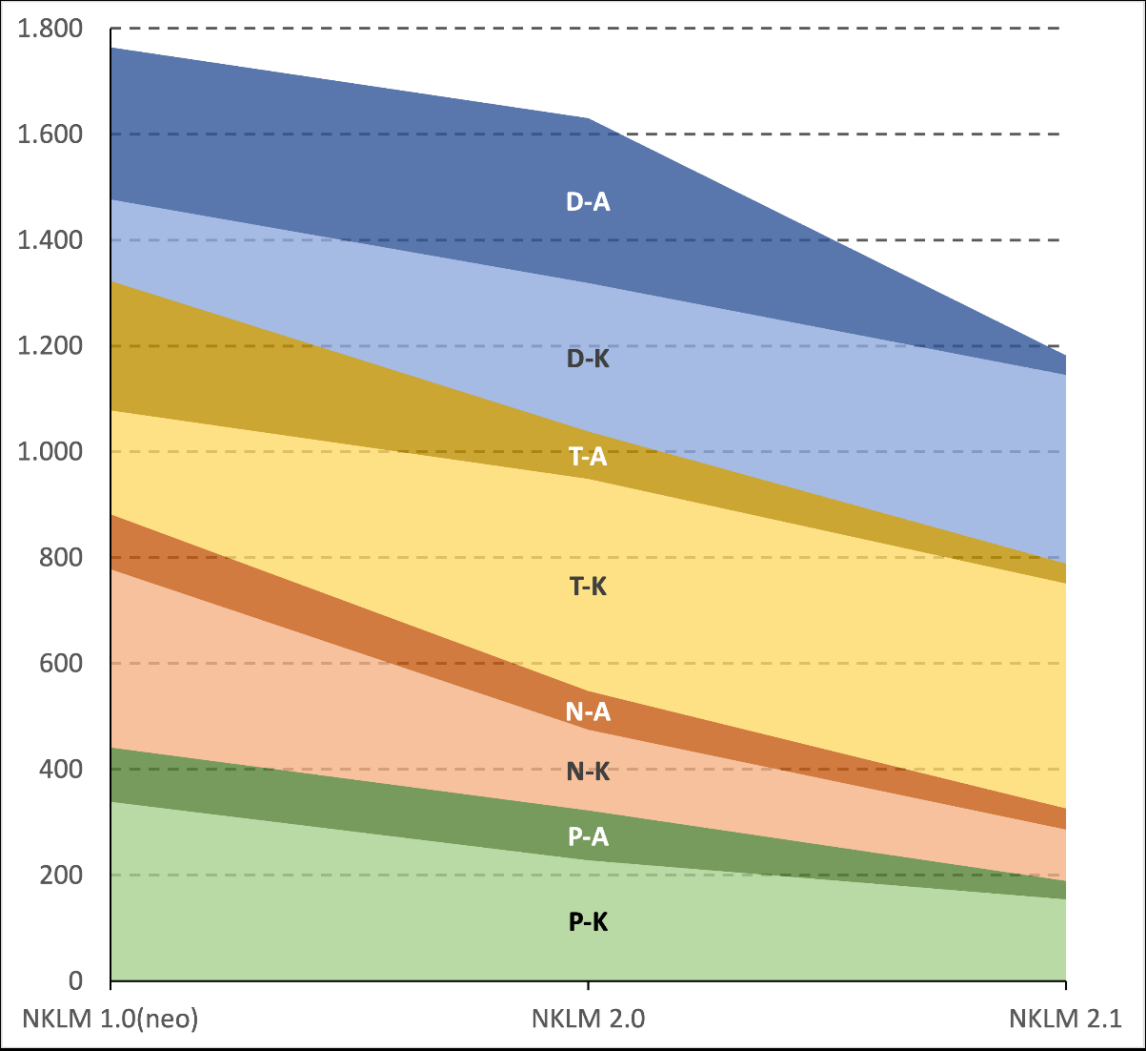
Absolute number of descriptors set across the three catalog versions NKLM 1.0 (neo), NKLM 2.0, and NKLM 2.1 For each descriptor set, the number of knowledge (K) and competences to act (A) is indicated. D (blue): Diagnostics, T (yellow): Therapy, N (orange): Emergency Measures, P (green): Prevention/Rehabilitation

Despite these increases in the number of diseases (especially in NKLM 2.0), the absolute number of descriptors set decreased across the catalogue versions, with the reduction varying in severity depending on the descriptor (see figure 3 (Fig. 3)).

**Figure 4:**
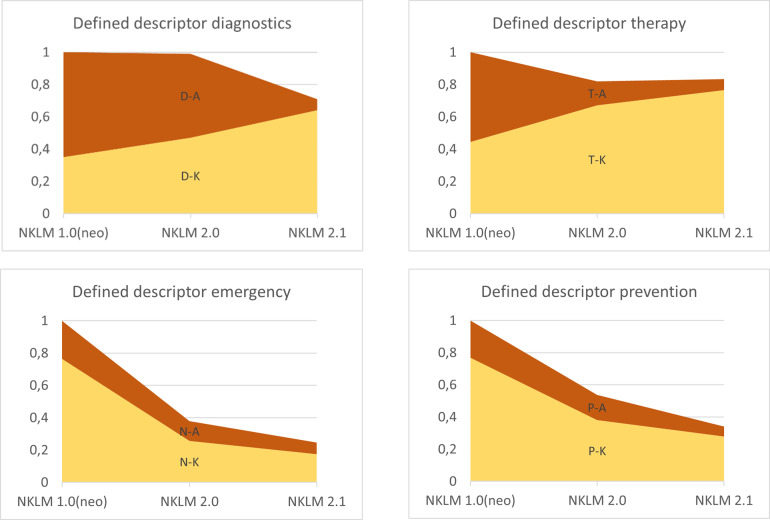
Relative number of descriptors set across the three catalog versions NKLM 1.0 (neo), NKLM 2.0, and NKLM 2.1 For each descriptor used, the proportion of knowledge (K) and action skills (A) is specified

After standardizing the descriptors set in terms of the number of diseases, a different pattern of reduction was observed for the individual descriptors, as shown in figure 4 (Fig. 4). All descriptors had significant differences in frequency across the catalogue versions in the chi-square test (p<0.001 everywhere, Cramer’s V for D: 0.4232; for T: 0.3529; for N: 0.4433; for P: 0.3818). 

### 3.3. Number of cross-links and completeness of the network of descriptors and cross-links

**Figure 5:**
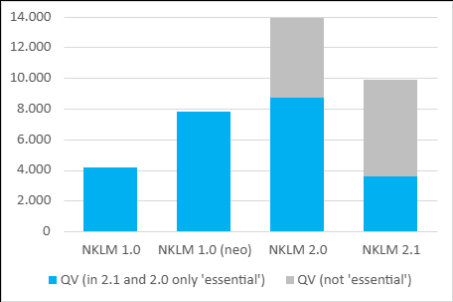
Absolute number of cross-references across catalog versions NKLM 1.0, NKLM 1.0 (neo), NKLM 2.0, and NKLM 2.1

**Figure 6:**
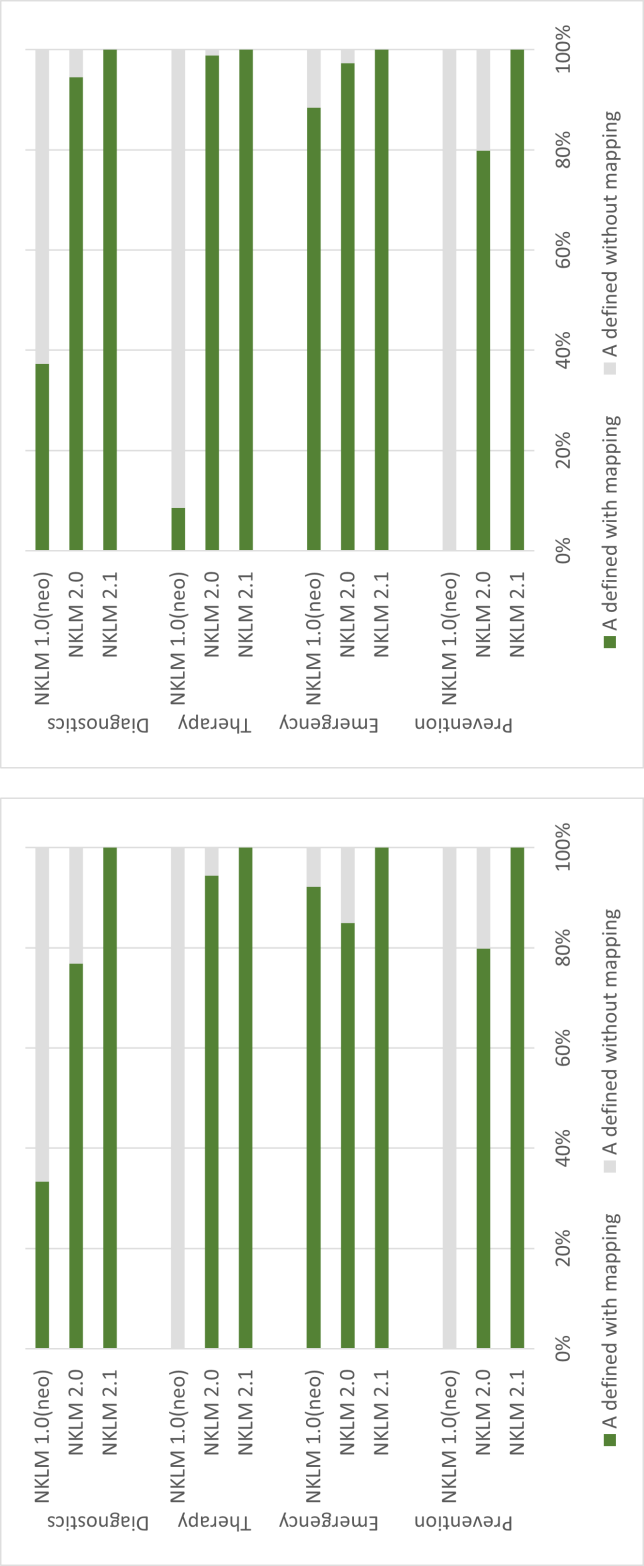
Proportion of A (competence to act) set across the three catalog versions NKLM 1.0 (neo), NKLM 2.0, and NKLM 2.1 For each descriptor type, the proportion of coverage by cross-references is indicated. Left: variant 1 (only cross-references to the target chapters), right: variant 2 (additional cross-references to other chapters)

For a better overview, the number of CR is also shown for NKLM 1.0 in figure 5 (Fig. 5). The number increased across the catalogue versions, but was reduced back below the initial level of NKLM 1.0 by defining the essential CR in NKLM 2.1. At the same time, the congruence of the set descriptors with the assigned CR was gradually increased up to NKLM 2.1. Figure 6 (Fig. 6) shows this for the descriptors with depth A, as only these were explicitly set in NKLM 1.0 (neo) and therefore only these are comparable across all catalogue versions. It can be seen that there were still large gaps in both NKLM 1.0 (neo) and NKLM 2.0, both in variant 1 (only CR to the target chapter) and in variant 2 (additional consideration of CR to other chapters). The results of the chi-square test for all descriptors of the different catalogue versions are shown in table 1 (Tab. 1).

**Figure 7:**
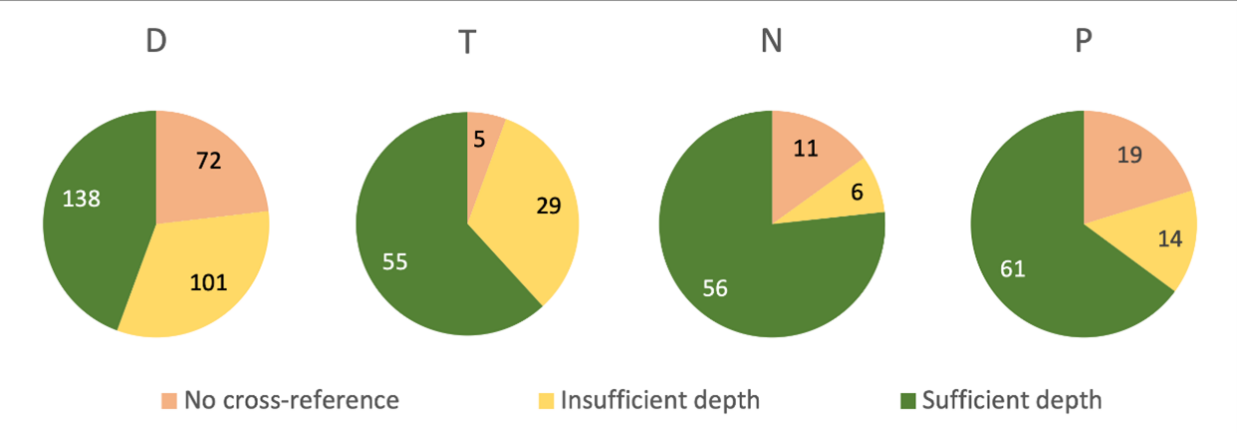
Number of descriptors set in NKLM 2.0 for diseases with missing cross-refrerences, cross- refrerences of insufficient depth, and cross-refrerences of sufficient depth D: Diagnostics, T: Therapy, N: Emergency Measures, P: Prevention/Rehabilitation

In addition, figure 7 (Fig. 7) shows that within NKLM 2.0, not only were many CR completely missing from the target chapters despite the descriptors set, but also that even where CR did exist, the depth A required for congruence with the descriptor was often not achieved. Both points are now 100% correctly represented in NKLM 2.1.

## 4. Discussion

To facilitate adoption of the NKLM by medical faculties [11], [15], two actions had to be taken: first, the total number of descriptors and CR needed to be reduced. Second, the learning content for each disease needed to be clearly defined [6], which required filling gaps in the reference network by a targeted, selective increase in the number of CR. The latter, in turn, required filling the gaps in the network, i.e., a targeted, selective increase in the number of CR. Overall, the results of this work indicate that the interventions were successful in terms of these two seemingly contradictory goals throughout the various process steps. The results are discussed below.

### 4.1. Conversion of free text into cross-references for NKLM 1.0 (neo)

The conversion of the text-based examples in NKLM 1.0 into actual CR led to a significant increase in CR within NKLM 1.0 (neo). This closed many gaps in the CR between the individual chapters. However, even after this optimization, there were still no corresponding CR in the respective target chapters for many assigned As. Thus, for many assigned descriptors, it was still unclear which specific content they were meant to represent.

### 4.2. Number of descriptors set

The reduction in descriptors described above may be due to various effects, as the individual interventions had different impacts on the respective descriptors and their depth of competence. For example, due to the introduction of CR descriptors for clearly defined target chapters, many LO had to be moved between chapters, adapted, or recreated in order to enable CR that complied with the rules. Overall, this necessary review of the LO affected led to the experts discussing the initially intended content more critically than before, resulting in a reduction in descriptors. This was facilitated by the division of disease groups, which is the main reason for the increase in the number of diseases, as it allowed for a more differentiated marking of competence.

In the case of diagnostics and therapy, contrary to the overall trend, an increase in the depth of competence knowledge can be observed. However, in most cases, no additional descriptors were marked, but rather, as part of the more critical expert discussion and the rule-related adaptations, previously set competence to act was reduced to knowledge. From the authors’ point of view, this has the following reasons:

1. A CR-LO must at least demonstrate the required depth of competence described in the descriptor. Although it would have been possible to raise the depth of competence of the CR-LO to competence to act, adapting the LO generally presents a greater hurdle than marking a descriptor, as the intended content must be explicitly described there.
2. In NKLM 2.1, general medical history taking and physical examination were no longer considered sufficient justification for marking a diagnostic descriptor. As a result, the corresponding cross-references were removed, although they had previously accounted for a large proportion of the competencies classified as “competence to act”.

In the case of the descriptors emergency measures and prevention/rehabilitation, there has been a particularly sharp decline in marked descriptors. A likely reason for this is the implicit setting of all descriptors with the competence depth of knowledge in NKLM 1.0 (neo). Apparently, emergency measures and prevention/rehabilitation were less relevant for students after detailed discussion among experts (if there were any emergency or prevention aspects to the respective disease at all).

At first glance, the shift from practical skills to knowledge in diagnostics and therapy described above contradicts the intention of the above mentioned master plan for Medical Education 2020 [8], which calls for more practical relevance in medical studies. However, the opposite is true: the elimination of descriptors (and associated cross-references) for basic medical skills simplifies practical learning because it can potentially take place in the context of any disease. This also facilitates curriculum development. 

In the context of the above-mentioned critical expert discussion, when marking descriptors with knowledge competence, it was explicitly questioned whether students should master procedures more theoretically or actually be able to perform them themselves.

### 4.3. Number of cross-references and completeness of the network of descriptors and cross-references

During the development of NKLM 1.0 and 2.0, some experts misunderstood the catalogue rules and created CR as non-binding examples rather than as mandatory entries. This contributed to the inhomogeneity of the CR. In the course of the interventions presented here, efforts were made to achieve homogenization of the catalogue reference structure through clear regulation. 

The separation between essential and non-essential CR greatly improved the clarity of the catalogue, as the latter must be displayed separately on the online platform if necessary. Essential CR were thus created according to clear guidelines and are presumably also of higher quality, as the individual CR were scrutinized more closely by the experts. 

The definition of target chapters and the resulting narrower definition of the content descriptor justifications has resulted in a further refinement of the CR structure, which has contributed to a reduction in the total number of CR. This can be seen from the fact that the total number of CR (even without separating them into essential and non-essential) decreased from NKLM 2.0 to NKLM 2.1.

### 4.4. Limitations

When creating NKLM 1.0 (neo), differences in understanding of terms between individuals may have influenced the mapping results. However, the rules developed on the basis of the pilot study should have contributed to a certain degree of objectivity.

The effects of the interventions carried out in the course of the NKLM revisions may also have been influenced by unrelated circumstances. For example, the working method for NKLM 2.1 has changed, as work was now carried out across chapters, enabling the experts to better understand the content networks.

Finally, it should be mentioned that the further development process for NKLM 2.1 has not yet been fully completed. However, based on the rules described, only minor adjustments to the descriptors or CR are to be expected.

## 5. Conclusions and outlook

The intention already formulated for NKLM 1.0 to transparently define *what a newly licensed physician should really know and be able to do, e.g., with regard to diabetes mellitus* [5] has been formally achieved with NKLM 2.1. This is also in line with the mentioned master plan for Medical Education 2020, which calls for teaching to be *geared toward the transfer of physician-related skills* [8]. 

The NKLM 2.1 is scheduled to be published in 2026, which will be followed by a further revision process to NKLM 3.0 and will hopefully bring further improvements. Future research projects should examine the feasibility and manageability of the NKLM at the faculties.

## Acknowledgements

The authors would like to thank all the experts involved in the various versions of the NKLM, who invested a great deal of (leisure) time in developing the catalogue. We would also like to thank all members of the MFT committees (especially the spokespersons of the NKLM coordination group) and all employees of the MFT office, who are working with great commitment to improve the catalogue. We would also like to thank all the staff of the LOOOP research and support team at Charité and MHB, as well as the many colleagues within the international LOOOP network for Research in Health Sciences Education, without whose ideas and support the development described here would not have been possible.

## Authors

### Equivalent authorship

- Till Rech and Jacqueline Jennebach contributed equally.
- Simon Drees and Olaf Ahlers contributed equally.

### Authors’ ORCIDs

- Till Rech: [0000-0002-7451-9038]
- Jacqueline Jennebach: 80009-0006-1572-8725]
- Martin R Fischer: [0000-0002-5299-5025]
- Felix Balzer: [0000-0003-1575-2056]
- Firman Sugiharto: [0000-0002-6874-5549]
- Martin Dittmar: [0000-0002-4288-564X]
- Vincent Wyszynski: [0009-0007-2157-4352]
- Olaf Fritze: [0000-0002-3825-3703]
- Simon Drees: [0000-0003-2693-8796]
- Olaf Ahlers: [0000-0003-1528-7182]

## Competing interests

The authors declare that they have no competing interests.

## Figures and Tables

**Table 1:**
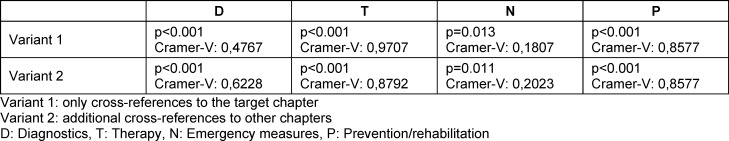
Results of chi-square test to verify the coverage of the set competence to act by required cross-references (see fig. 6)
